# The primary therapy chosen for patients with localized prostate cancer between the university hospital and its affiliated hospitals in Nara Uro-oncological research group registration

**DOI:** 10.1186/1471-2490-11-6

**Published:** 2011-04-27

**Authors:** Nobumichi Tanaka, Kiyohide Fujimoto, Akihide Hirayama, Shoji Samma, Hitoshi Momose, Yoshiteru Kaneko, Masaki Haramoto, Yoshiki Hayashi, Yoshinori Nakagawa, Takeshi Otani, Shuji Watanabe, Yoshihiko Hirao

**Affiliations:** 1 Department of Urology, Nara Medical University, Kashihara, Japan; 2 Nara Uro-Oncological Research Group, Kashihara, Japan

## Abstract

**Background:**

We investigated the differences between the preferential primary therapy conceived by the primary doctors and the primary therapy actually conducted for prostate cancer patients in Nara, Japan.

**Methods:**

The distribution of primary therapy and clinical characteristics of 2303 prostate cancer patients - diagnosed between 2004 and 2006 at Nara Medical University and its 23 affiliated hospitals - were assessed. Moreover, the preferential primary therapy for the patients at each clinical stage (cT1-T3bN0M0) conceived by the primary doctors was investigated and compared to the actual therapy.

**Results:**

Of all patients, 51% received primary androgen deprivation therapy (PADT), 30% underwent radical prostatectomy (RP), and 14% received radiation therapy (RT). The preferential primary therapy for cT1-2N0M0 was RP (92%) while 38% of the patients actually received PADT (RP: 40%). For cT3aN0M0, the preferential primary therapy was both RP and external beam radiation therapy (EBRT) while 58% of the patients actually received PADT (RP: 16%, EBRT: 24%). For cT3bN0M0, the most preferential primary therapy was EBRT (46%) while 67% of the patients actually received PADT (EBRT: 21%). This trend was more notable in the affiliated hospitals than in the University hospital. The hospitals with lower volume of RP per year significantly conducted PADT compared with those with higher volume of RP.

**Conclusions:**

PADT was commonly used to treat localized prostate cancer as well as locally advanced prostate cancer in Japan. There was a definite discrepancy between the preferential primary therapy conceived by the primary doctors and the actual therapy provided to the patients.

## Background

The distribution of the primary therapies for prostate cancer is different between USA and Japan. Specifically, more patients receive definitive therapies in USA than in Japan. The CaPSURE data from USA indicated that 44% of patients underwent radical prostatectomy (RP), 23% received definitive radiotherapy (external beam radiation therapy: EBRT, and brachytherapy: BT), and 20% received primary androgen deprivation therapy (PADT) [1]. On the other hand, our data from Nara Uro-Oncological Research Group (NUORG) showed that the corresponding figures were 30%, 14%, and 51%, respectively [2]. The differences are outstanding, particularly in the low-risk group with organ-confined stage and Gleason score of <7 and PSA value of <10 ng/mL. Analysis of the CaPSURE data between 2004 and 2006 [3] revealed that 60% of the low-risk patients received RP and 7% received PADT. In contrast, 43% and 27% of our low-risk patients received RP and PADT, respectively [2]. The higher rate of hormonal therapy as the primary therapy among the Japanese prostate cancer patients is remarkable and unique when compared with American and European counterparts.

The contemporary trend of selecting the primary therapy for prostate cancer varies among countries and races according to the different socioeconomic situations. Reportedly the African American and low-income patients are very apt to undergo RP and receive radiotherapy of the relatively low medical cost [4,5]. The elderly (over 75 years) and the patients with high academic background showed negative correlation with the primary hormonal therapy and positive correlation with the primary radiotherapy [6]. Ethnicity closely correlated with the rate of choosing curative therapies [7]. The African Americans were more likely to receive radiotherapy as compared with RP, while Latinos were less likely to receive radiotherapy as compared with RP. Taken together, several socioeconomic factors (e.g., age, ethnicity, educational level, and medical insurance system) are very likely to influence the choice of primary therapy for prostate cancer.

In this study, we investigated the trends of the most preferential primary therapy conceived by urologists and the primary therapy actually provided for patients with localized prostate cancer in our NUORG institutions. We then evaluated the differences in the primary treatment choice between the university hospital and its affiliates.

## Methods

A total of 2371 patients diagnosed with prostate cancer at Nara Medical University (NMU) hospital and its 23 affiliated hospitals between January 2004 and December 2006 were surveyed in this study. Due to incomplete information at diagnosis, 68 patients were excluded from this study. In total, 2303 patients were evaluated for the clinical TNM classification of 2002 UICC, Gleason score of biopsy specimen, PSA value measured at initial diagnosis, and actual primary therapy.

We asked 24 instructive urologists certificated by the Japanese Urological Association (JUA) in all 24 hospitals about the primary therapy for localized prostate cancer in each stage (namely, cT1-2N0M0, cT3aN0M0 and cT3bN0M0) using a self-developed questionnaire. The questionnaire included questions about the most preferential primary therapy which they conceived if a given patient had no concomitant diseases. As a treatment option, PADT, RP, EBRT, BT (high-dose-rate and low-dose rate) and watchful waiting (WW) were included in this self-developed questionnaire. These modalities are covered by public health insurance system in Japan. On the other hand, cryosurgery and High Intensity Focused Ultrasound are not covered by public health insurance system, and therefore, these treatment options were not included in this questionnaire. The distribution of the most preferential primary therapy for localized and locally advanced prostate cancer conceived by the urologists was compared to the chosen and performed primary therapy. Furthermore, we compared the real status of primary therapy between the university hospital and its affiliated hospitals.

To elucidate the correlation between the number of RP per year and the proportion of PADT, we divided all hospitals by 3 groups (Group 1, RP: 10 cases or less per year; Group 2, 11-30 cases per year; and Group 3, greater than 30 cases per year). The distribution of primary therapy among these 3 groups was examined, and Spearman's rank correlation test was used to examine the significance of correlation between the number of RP per year and the distribution of PADT at each group.

To analyze the differences in categorical parameters, the chi-square test was employed. Mann-Whitney U test was used to evaluate the differences in continuous variables. All statistical analyses were performed using PASW Statistics 17.0 (SPSS Inc., Chicago, IL, USA). All *p *values less than 0.05 were considered as statistically significant.

The institutional reviewer board approved this retrospective study. Obtaining informed consent from the patients was exempted in the respect of the aim and methods of this study.

## Results

### Primary therapy

The demographic characteristics of all 2303 patients are shown in Table 1. The mean values of the patients' age and PSA value at diagnosis were 71.8 (median: 72.0) years and 137.5 (median: 12.2) ng/mL, respectively. The median age and PSA value at diagnosis in the affiliated hospitals were significantly higher than those in the university hospital (age: 73.0 vs. 71.0 years, *p *< 0.001; PSA: 13.1 vs. 9.6 ng/mL, *p *< 0.001, respectively). Of all 2303 patients, 2117 (91.9%) showed no regional nodal metastasis, 142 (6.2%) had regional nodal metastasis, and 44 were not informative about the nodal status. Furthermore, 1972 (86.6%) showed no distant metastasis, 84 (12.4%) had distant metastasis (11 with M1a, 257 with M1b, and 16 with M1c), and 47 were not informative. The patients who were treated at the university hospital were younger with lower PSA level, lower Gleason score, and lower clinical T stage (*p *< 0.001).

**Table 1:** Demographic characteristics of 2303 patients

	Overall	University hospital	Affiliated hospitals	
			
	n = 2303 (%)	n = 420 (%)	n = 1883 (%)	*P *value
*Age (years)*				
younger than 60	154 (6.7)	52 (12.4)	102 (5.4)	
60-69	684 (29.7)	130 (31.0)	554 (29.4)	
70-79	1117 (48.5)	199 (47.3)	918 (48.8)	
80 or older	348 (15.1)	39 (9.3)	309 (16.4)	< 0.001
				
*PSA at diagnosis*				
10.0 or less	963 (41.8)	225 (53.5)	738 (39.2)	
10.1-20	554 (24.1)	98 (23.3)	456 (24.2)	
greater than 20	786 (34.1)	97 (23.1)	689 (36.6)	< 0.001
				
*Gleason score*				
2-6	906 (39.3)	146 (34.8)	760 (40.3)	
7	722 (31.4)	175 (41.6)	547 (29.0)	
8-10	675 (29.3)	99 (23.6)	576 (30.6)	< 0.001
				
*Clinical T stage*				
T1	766 (33.3)	183 (43.6)	583 (31.0)	
T2	933 (40.5)	134 (31.9)	799 (42.4)	
T3	489 (21.2)	95 (22.6)	394 (20.9)	
T4	115 (5.0)	8 (1.9)	107 (5.7)	< 0.001

Chi-square test.

Among the 2303 patients, 51% were treated with PADT, 30% underwent RP, 14% received radiation therapy (RT), and 2% chose WW as the primary therapy. The proportion of PADT in the affiliated hospitals was significantly higher than that in the university hospital (57% vs. 24%). The proportion of RP was comparable between the university hospital and the affiliated hospitals (29% vs. 31%). The proportion of EBRT in the university hospital was significantly higher than that in the affiliated hospitals (44% vs. 7%) (*p *< 0.001).

### Preferential primary therapy

**Figure 1:**
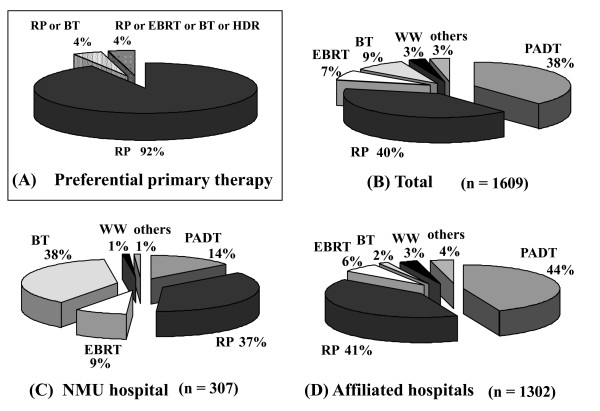
**The distribution of the preferential primary therapy (A), the actual primary therapy for all 1609 patients (B), in NMU hospital (C), and in the affiliated hospitals (D) for the patients with cT1-2N0M0 (Chi-square test; = 0.001, NMU hospital vs. affiliated hospitals)**. NMU; Nara Medical University, RP; radical prostatectomy, PADT; primary androgen deprivation therapy, HDR; high dose rate brachytherapy, BT; brachytherapy, EBRT; external beam radiation therapy, WW; watchful waiting.

Of the 2303 patients, 1609 were in cT1-2N0M0, 211 were in cT3aN0M0, and 94 were in cT3bN0M0. Regarding the preferential primary therapy of cT1-2N0M0, RP was highly recommended by urologists (92%). In contrast, no urologist raised PADT as the preferential primary therapy (Figure 1A). With regard to the actual therapies, however, only 40% of patients underwent RP and 38% received PADT (Figure 1B). The number of patients who received PADT in the affiliated hospitals was higher when compared to that in the university hospital (44% vs. 14%, *p *= 0.001) (Figure 1C, D).

**Figure 2:**
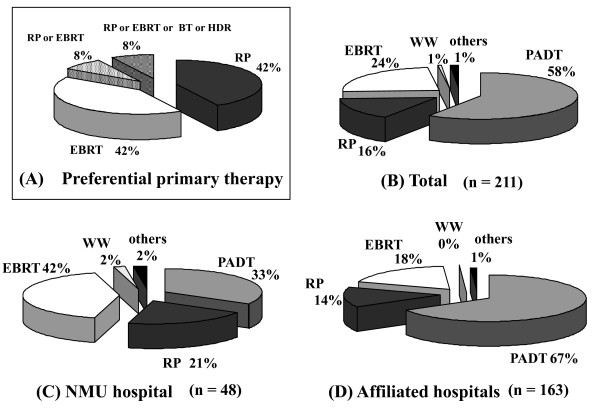
**The distribution of the preferential primary therapy (A), the performed primary therapy for all 211 patients (B), in NMU hospital (C), and in the affiliated hospitals (D) for the patients with cT3aN0M0 (Chi-square test; < 0.001, NMU hospital vs. affiliated hospitals)**. NMU; Nara Medical University, RP; radical prostatectomy, PADT; primary androgen deprivation therapy, HDR; high dose rate brachytherapy, BT; brachytherapy, EBRT; external beam radiation therapy, WW; watchful waiting.

Concerning the breakdown of the preferential primary therapy for cT3aN0M0, the proportions of RP and EBRT were equal (42% each) (Figure 2A). Interestingly, breakdown of the actual therapies revealed that 58% of the patients received PADT (Figure 2B). The proportion of PADT in the affiliated hospitals was 67%, being significantly higher than in the university hospital (33%, < 0.001) (Figure 2C, D). Conversely, the proportion of EBRT in the university hospital was significantly higher than in the affiliated hospitals (42% vs. 18%, *p *< 0.001).

**Figure 3:**
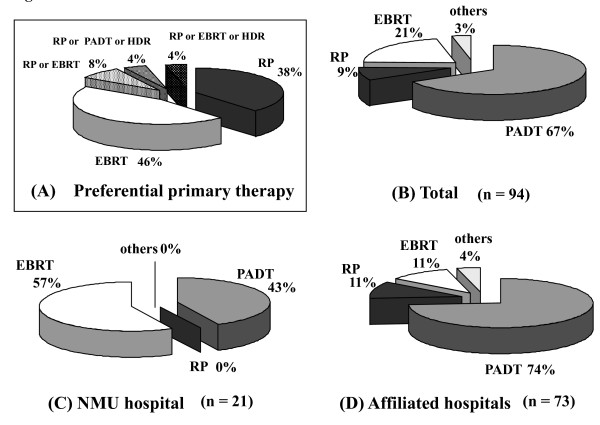
**The distribution of the preferential primary therapy (A), the performed primary therapy for all 94 patients (B), in NMU hospital (C), and in the affiliated hospitals (D) for patients with cT3bN0M0 (Chi-square test; < 0.001, NMU hospital vs. affiliated hospitals)**. NMU; Nara Medical University, RP; radical prostatectomy, PADT; primary androgen deprivation th1erapy, HDR; high dose rate brachytherapy, EBRT; external beam radiation therapy, WW; watchful waiting.

Regarding the preferential primary therapy for cT3bN0M0, RP and PADT were adopted by 38% and 46% of urologists, respectively (Figure 3A). In contrast, the proportions of the actual therapies were 67% for PADT, 21% for EBRT, and 9% for RP (Figure 3B). In the affiliated hospitals, 74% of the patients received PADT whereas 43% received PADT in the university hospital (*p *< 0.001) (Figure 3C, D). The proportion of EBRT in the university hospital was significantly higher than that in the affiliated hospitals (57% vs. 11%, *p *< 0.001).

### The correlation between the number of RP and the distribution of PADT

**Figure 4:**
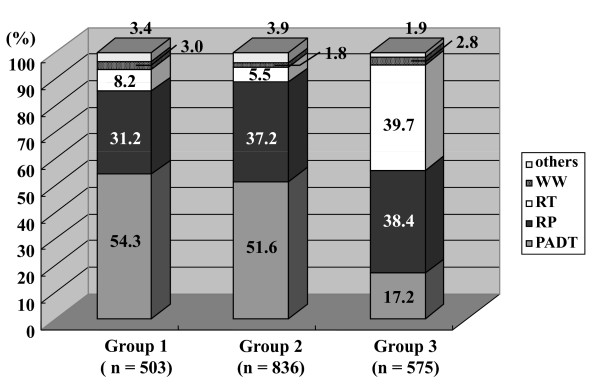
**The distribution of the primary therapy at each hospital group in patients with cT1-3N0M0 (Chi-square test; < 0.001,)**. Group 1; The hospitals with 10 RP cases or less per year. Group 2; The hospitals with 11 to 30 RP cases per year. Group 3; The hospitals with greater than 30 RP cases per year. RP; radical prostatectomy, PADT; primary androgen deprivation therapy, RT; radiation therapy, WW; watchful waiting.

Of the 2303 patients, 1914 were in cT1-3N0M0. Of all hospitals, 15 hospitals were defined as Group 1 (the number of RP per year: 10 cases or less), 7 hospitals as Group 2 (the number of RP per year: 11 to 30 cases), and 2 hospitals as Group 3 (the number of RP per year: greater than 30 cases). The University hospital is in Group 3, and RT is available in both hospitals in Group 3. The distribution of primary therapy was significantly different among these 3 groups (Figure 4, *p *< 0.001). The group with higher percentage of RP showed significantly lower percentage of PADT compared with the group with lower percentage of RP. Moreover, there was a significant inverse correlation between the number of RP and the distribution of PADT (r = -0.291, *p *< 0.001).

## Discussion

Previously we reported that our series of patients (treated between 2004 and 2006) received PADT more frequently than in USA [2]. Relatively more patients chose PADT as the primary therapy not only for locally advanced prostate cancer but also for localized prostate cancer in Japan [8-11]. This nation-wide trend in Japan is apparently dissimilar from that in the Western countries [1-3]. According to the JUA report, for example, 57% of the registered patients received PADT, 28% underwent RP with or without neoadjuvant or adjuvant therapy, and 8% received RT with or without neoadjuvant therapy in 2000 [9]. In comparison between our report and the 2000 JUA report, the proportion of RT in our report was significantly higher than that of JUA report (14% vs. 8%), while the proportion of PADT was significantly lower than that of the JUA report (51% vs. 57%) (*p *< 0.001, chi-square test; data not shown). Since low-dose rate brachytherapy was legally approved by the government in 2003, RT overall including high-quality EBRT like intensity modulated radiation therapy and three-dimensional radiation therapy has been increasing no less than brachytherapy in Japan year after year. The proportion of RT is likely to hereafter increase alternatively to PADT in Japan.

Several specific reasons for this trend in Japan are proposed. First, all patients are covered completely by the total public health insurance system in Japan. All patients including even no-income people can receive any therapy they like under the public insurance coverage. In principle, the patients continue PADT until they show androgen independency. The therapy cost increases until cessation of PADT. Secondly, the Japanese patients tolerate well receiving hormonal therapy without severe side effects. Akaza *et al *reported that there was no significant difference in the overall survival between the patients with localized prostate cancer treated with PADT and the age-matched men in the general population [12]. Akaza *et al *also reported that the incidence of cardiovascular events in patients treated with leuprorelin was not greater than that expected in the general Japanese population [13]. On the other hand, the newly diagnosed prostate cancer patients who received PADT for at least one year showed a 20% higher risk of serious cardiovascular morbidity as compared with similar men who did not receive PADT in the SEER database [14]. Although there is no direct comparison of the cardiovascular morbidity in the patients who receive PADT between Japanese and Western men, serious cardiovascular events are apparently not frequently experienced in the Japanese patients who received hormonal therapy. Thirdly, the majority of the Japanese patients interestingly do not reject receiving hormonal therapy because of the low priority of preserving erectile function at the time of treatment selection. Additionally, most recently robotic surgery was introduced in Japan and robotic-assisted radical prostatectomy is currently available in the several legally authorized hospitals. Since the number of robotic-assisted radical prostatectomy cases has belatedly increased in those limited institutes, the near-future prevalence of this surgical modality will not a little influence selection of less invasive primary therapy for early prostate cancer.

In our data, most primary urologists conceived that RP is the preferential primary therapy for cT1-2N0M0 prostate cancer. Nevertheless, the proportion of patients who actually chose and received RP was 40%, but those who received PADT accounted for nearly 40%. Of course, some patients could not receive the definitive therapy due to several limitations such as co-morbidity, old age and so on. Nonetheless, the proportion of PADT for this clinical stage was higher in Japan than in the Western countries [3]. Since only 3 affiliated hospitals could provide EBRT, and BT is currently available only in the university hospital, this status strongly affects the different proportions of EBRT and BT between the university hospital and its affiliates. Indeed, the present high proportion of BT and EBRT (over 45%) was attributed to the recent increase in patients who were referred to the university hospital with their own choice for RT.

RP and EBRT were equally selected (42% each) as the preferential primary therapy for cT3aN0M0 prostate cancer. Interestingly, over 40% of urologists considered RP as the preferential therapy for cT3a prostate cancer. However, PADT was the highest with the actual rate of 58%. In the university hospital, 42% of the patients received EBRT and 33% underwent PADT. On the other hand, two-thirds of patients received PADT in the affiliated hospitals. Although 46% of urologists selected EBRT for cT3bN0M0, an unexpectedly high rate (38%) of urologists considered that RP was still the preferential primary therapy. However, two-thirds of the patients actually received PADT while 21% and 9% received EBRT and RP, respectively. In the university hospital, no patients with cT3b prostate cancer underwent RP.

Overall, the proportion of PADT in our data was as high as that of the JUA report [9]. Relatively a large population of the urologists is likely to choose RP as the preferential primary therapy for cT3 stage. However, the most common primary therapies were RT in the university hospital and PADT in its affiliated hospitals. A small number of the urologists actually still performed RP even for higher stage prostate cancer. Our speculation is that this trend in the affiliated hospitals may be due to retrospection of unpleasant experiences with old-fashioned RT and unawareness of the recent technological advances in RT during their long-term career as urologists. The educational and institutional prevalence of RT, as an alternative to PADT, seems to be necessary for both urologists and patients with advanced prostate cancer in Japan.

Interestingly, the present study showed that the hospitals with lower volume of RP significantly conducted PADT compared with those with higher volume of RP in patients with cT1-3N0M0 (Figure 4). RT was available in 1 hospital in Group 2 and all hospitals in Group 3. The proportion of PADT was significantly higher in the hospitals where RT was not available in own hospital than those where RT was available. Recently, Barocas *et al *reviewed the available literature on the impact of surgeon and hospital volume on patient outcomes after RP. They reported a substantial advantage (e.g. length of hospital stay, peri-operative complications, peri-operative mortality, treatment failure and so on) for patients treated in high volume hospitals and by high volume surgeons [15]. It is conceivable that the choice of primary therapy by patients and doctors would depend on the volume of RP and the variety of treatment modalities. Unfortunately, the correlation between high volume hospital and the outcomes in the present study cohort has not been evaluated. A further investigation with substantial follow-up period makes it possible to elucidate this issue.

There were several limitations of this study. First, we used a self-developed questionnaire to determine the preferential therapy by doctors at each clinical stage. This questionnaire has not been externally validated. Second, we did not conduct the survey in patients stratified risk classification. It is more preferable to survey the preferential primary therapy at the moment. Third, this study was lacking in the aspect of co-morbidity of patients. Fourth, it would be more informative if a patient-directed questionnaire were adopted.

## Conclusions

There are explicit differences in choosing the primary therapy for prostate cancer between the university hospital and its affiliated hospitals, and PADT was commonly provided to our patients with localized prostate cancer as well as those with locally advanced prostate cancer in the affiliated hospitals. This trend was mainly caused by discrepancy between the preferential primary therapies conceived by urologists and the actual treatment after thorough considerations of the patients' backgrounds and available therapeutic modalities.

## Competing interests

The authors declare that they have no competing interests.

## Authors' contributions

All authors made substantial contributions to the acquisition and interpretation of data, critical revision of the manuscript for important intellectual content, and approved the final version for publication. YH made substantial contributions to the conception and design of the study. NT performed the statistical analysis

## Pre-publication history

The pre-publication history for this paper can be accessed here:

http://www.biomedcentral.com/1471-2490/11/6/prepub
